# A literature review and meta-analysis of safety profiles of SGLT2 inhibitors in Japanese patients with diabetes mellitus

**DOI:** 10.1038/s41598-021-92925-2

**Published:** 2021-06-29

**Authors:** Junichi Mukai, Shinya Kanno, Rie Kubota

**Affiliations:** Division of Clinical Pharmacy (Laboratory of Clinical Pharmacy Education) and Research and Education Center for Clinical Pharmacy, School of Pharmacy, Kitasato University, 5-9-1 Shirokane, Minato-ku, Tokyo, 108-8641 Japan

**Keywords:** Endocrinology, Health care, Medical research

## Abstract

The safety profiles of sodium-glucose co-transporter 2 (SGLT2) inhibitors may depend on races/ethnicities. We aimed to assess the safety profiles of SGLT2 inhibitors in Japanese patients with diabetes mellitus (DM). The electronic databases MEDLINE, CENTRAL, and Ichushi-web were searched for studies with no language restriction from their inception to August 2019. Trials were included in the analysis if they were randomized controlled trials (RCTs) comparing the effects of SGLT2 inhibitors with a placebo in Japanese patients with DM > 18 years and reporting HbA1c and at least 1 adverse event. We calculated risk ratios with 95% CIs and used a random-effects model. Of the 22 RCTs included in our review, only 1 included patients with type 1 DM. The durations of RCTs ranged between 4 and 24 weeks. In comparison with a placebo, SGLT2 inhibitors were associated with similar risks of hypoglycemia, urinary tract infection, genital infection, hypovolemia, and fracture. The outcomes of treatment with SGLT2 inhibitors among Japanese patients with DM suggest favorable safety profiles. However, further evidence from studies with a longer duration, involving more diverse populations, such as patients with different types of DM, or including individual SGLT2 inhibitors is needed to resolve the limitations of the present study.

## Introduction

Sodium-glucose co-transporter 2 (SGLT2) inhibitors are novel oral hypoglycemic agents that exert beneficial effects on glycemic control and weight loss in patients with type 1 and 2 diabetes mellitus (DM)^1–3^. To date, SGLT2 inhibitors have been shown to exert these effects among different races/ethnicities. For example, recent randomized controlled trials (RCTs), which included approximately 80% Caucasians, showed favorable effects on glycemic control and weight loss as well as in Asians^4–6^. Furthermore, meta-analyses recently showed that the effects of SGLT2 inhibitors on not only glycemic control and weight loss, but also lipid profiles were similar between Asian and non-Asian patients^7,8^.

In contrast, previous findings on the safety profiles of SGLT2 inhibitors among different races/ethnicities have been inconsistent; one meta-analysis showed that the safety profiles of SGLT2 inhibitors differed between Asian and non-Asian patients. SGLT2 inhibitors increased the risk of urinary tract infection (UTI) in non-Asian patients with type 2 DM, but were associated with a similar risk of UTI as a placebo in Asian patients with type 2 DM^7^. This study also indicated that the risk of hypoglycemia was higher in non-Asian patients with type 2 DM who were treated with SGLT2 inhibitors than in Asian patients with type 2 DM^7^. Another study that focused on racial differences found that the risk of cough was higher in East Asian patients who are treated with angiotensin-converting enzyme inhibitors than in Caucasian patients^9^. Based on these findings, we hypothesized that the safety profiles of SGLT2 inhibitors in patients with DM depend on racial differences. Moreover, to the best of our knowledge, no meta-analyses have examined the safety profiles of SGLT2 inhibitors in Japanese patients with DM. Therefore, we herein conducted a systematic review and meta-analysis to summarize the available literature and appraise the safety profiles of SGLT2 inhibitors in Japanese patients with DM.

## Methods

### Search strategies for the identification of RCTs

We searched the electronic databases MEDLINE, The Cochrane Central Register of Controlled Trials (CENTRAL), and Japana Centra Revuo Medicina (Ichushi-web) for studies from their inception to 26 August 2019. Information on 6 types of SGLT2 inhibitors currently approved in Japan was collected: canagliflozin (CANA), dapagliflozin (DAPA), empagliflozin (EMPA), ipragliflozin (IPRA), luseogliflozin (LUSEO), and tofogliflozin (TOFO). We used individual SGLT2 inhibitor names, alternative names, “sodium-glucose transporter 2”, and “SGLT2 inhibitors” as search terms. We restricted our search to “randomized controlled trial” in these electronic databases. A reference search was also implemented from relevant studies in order to identify more RCTs. The study search was undertaken independently by 2 authors (SK and JM). Any discrepancies were settled by discussions between the 2 assessors.

### Management for data extraction

We did not impose any language restriction. Trials were included if they were RCTs (1) comparing the effects of SGLT2 inhibitors with a placebo in Japanese patients with DM who were 18 years or older, and (2) reporting HbA1c and at least 1 adverse event. We excluded cross-over trials, RCTs with no information available on races/ethnicities, and RCTs involving healthy subjects. We extracted data on the types of DM, co-interventions as medication use, the daily dose of each SGLT2 inhibitor, and baseline profiles: HbA1c, body mass index (BMI), age, and the estimated glomerular filtration rate (eGFR). The safety outcomes of interest were as follows: hypoglycemia, UTI, genital infection, hypovolemia, fracture, and diabetic ketoacidosis. The term UTI included cystitis. Other definitions of safety outcomes were followed as defined by each author of the study.

### Quality assessment of each RCT

Study quality was rated using the Jadad scale and risk of bias tool. The Jadad scale is used to evaluate the appropriateness of the randomization technique, the method used for double-masking, and descriptions of dropouts or withdrawals^10^. The scale ranges between 0 and 5 points. We included studies that scored 4 points or higher in the analysis. The risk of bias for the studies was assessed using the Cochrane Collaboration’s tool^11^. Seven items were examined for the risk of bias: random sequence generation, allocation concealment, the blinding of participants and personnel, blinding of outcome assessments, incomplete outcome data, free of selective reporting, and quality evidence on safety parameters as other sources of bias. Each of the 7 items was scored as a “low risk”, “unclear risk”, or “high risk”.

### Data synthesis

We calculated the risk ratio with 95% CI for each safety outcome. The heterogeneity of each outcome was evaluated using chi-squared and I^2^ statistics. A value of 40% or more was defined to represent marked heterogeneity^11^. We used a random-effects model (the Mantel–Haenszel method^12^) to more conservatively assess outcomes. In the meta-analysis, multiple SGLT2 inhibitor groups in a single trial were combined into a single group^11^. Subgroup analyses were performed by including only patients with type 2 DM and only patients who were treated with a SGLT2 inhibitor as monotherapy. We drew a funnel plot to assess publication bias visually when there were 10 RCTs or more in the meta-analysis^11^. All statistical analyses were performed with review manager 5.3 software (The Nordic Cochrane Centre, The Cochrane Collaboration, 2014). A P value less than 0.05 was considered to be significant.

## Results

We identified 765 studies in the database search. One hundred and eighty-one full texts were retrieved after the removal of duplications and screening of titles and abstracts. Twenty-two RCTs were ultimately included in our review. Supplementary Figure S1 shows the process used to identify eligible RCTs^13–34^ following PRISMA^35^. Table 1 shows the characteristics of RCTs included in the meta-analysis. Only 1 study included patients with type 1 DM^21^. Six types of SGLT2 inhibitors were collected: CANA, DAPA, EMPA, IPRA, LUSEO, and TOFO. The durations of RCTs ranged between 4 and 24 weeks. Since 1 study had data on safety profiles at weeks 24 and 52^20^, we extracted the former data before the up-titration of EMPA was initiated. All trials were published in English.

**Table 1:** Characteristics of 22 randomized controlled trials included in the meta-analysis.

Author	Types of DM	Concomitant medications	Doses [mg/day]	N	Duration (weeks)	Age (years)^a^	HbA1c (NGSP, %)^a^	BMI (kg/m^2^)^a^	eGFR (mL/min/1.73 m^2^)^a^	Jadad scale^b^
Inagaki 2013^13^	Type 2	None	CANA 50 mg	82	12	57.4	8.13	25.11	83.5	5
			CANA 100 mg	74		57.7	8.05	25.61	86.9	
			CANA 200 mg	76		57.0	8.11	25.51	83.8	
			CANA 300 mg	75		57.1	8.17	25.89	86.9	
			Placebo	75		57.7	7.99	26.41	83.0	
Inagaki 2014^14^	Type 2	None	CANA 100 mg	90	24	58.4	7.98	25.59	81.4	5
			CANA 200 mg	88		57.4	8.04	25.43	87.2	
			Placebo	93		58.2	8.04	25.85	84.7	
Inagaki 2016^15^	Type 2	Insulin	CANA 100 mg	76	16	59.7	8.89	26.88	83.8	5
			Placebo	70		56.1	8.85	25.99	86.1	
Kadowaki 2017^16^	Type 2	Teneligliptin	CANA 100 mg	70	24	58.4	8.18	25.53	84.7	5
			Placebo	68		56.0	7.87	26.44	83.9	
Araki 2016^17^	Type 2	Insulin, DPP-4 inhibitor	DAPA 5 mg	122	16	58.3	8.26	26.89	NR	4
			Placebo	60		57.6	8.52	26.12	NR	
Kaku 2013^18^	Type 2	None	DAPA 1 mg	59	12	55.9	8.10	NR	NR	5
			DAPA 2.5 mg	56		57.7	7.92	NR	NR	
			DAPA 5 mg	58		58.0	8.05	NR	NR	
			DAPA 10 mg	52		56.5	8.18	NR	NR	
			Placebo	54		58.4	8.12	NR	NR	
Kadowaki 2014^19^	Type 2	Rescue therapy	EMPA 5 mg	110	12	57.3	7.92	26.3	86.5	4
			EMPA 10 mg	109		57.9	7.93	25.3	85.8	
			EMPA 25 mg	109		57.2	7.93	25.1	85.2	
			EMPA 50 mg	110		56.6	8.02	25.0	86.5	
			Placebo	109		58.7	7.94	25.6	84.6	
Kawamori 2018^20^	Type 2	Linagliptin, Rescue therapy	EMPA 10 mg	182	52 (24)*	60.0	8.27	26.0	89.3	5
			Placebo	93		59.8	8.36	26.6	86.3	
Shimada 2018^21^	Type 1	Insulin	EMPA 2.5 mg	13	4	44.2	8.02	24.4	88.0	5
			EMPA 10 mg	12		44.5	8.12	22.68	87.0	
			EMPA 25 mg	12		46.6	7.89	22.6	88.8	
			Placebo	11		43.9	8.23	23.7	95.1	
Ishihara 2016^22^	Type 2	Insulin, DPP-4 inhibitor	IPRA 50 mg	168	16	58.7	8.67	25.61	83.98	5
			Placebo	87		59.2	8.62	26.42	80.11	
Kashiwagi 2014^23^	Type 2	None	IPRA 12.5 mg	73	12	55.3	8.39	25.6	NR	4
			IPRA 25 mg	74		57.0	8.32	26.2	NR	
			IPRA 50 mg	72		55.9	8.33	25.8	NR	
			IPRA 100 mg	72		56.0	8.25	25.9	NR	
			Placebo	69		55.2	8.36	25.1	NR	
Kashiwagi 2015A^24^	Type 2	Sulfonylurea	IPRA 50 mg	165	24	59.6	8.38	25.81	84.24	5
			Placebo	75		59.8	8.34	24.18	85.87	
Kashiwagi 2015B^25^	Type 2	Pioglitazone	IPRA 50 mg	97	24	56.2	8.24	27.11	NR	5
			Placebo	54		56.1	8.39	27.13	NR	
Kashiwagi 2015C^26^	Type 2	None	IPRA 50 mg	62	16	60.6	8.40	25.3	NR	5
			Placebo	67		58.3	8.25	25.6	NR	
Kashiwagi 2015D^27^	Type 2	Antidiabetic agents	IPRA 50 mg	118	24	63.9	7.53	25.84	60.2	5
			Placebo	46		65.7	7.55	24.96	62.7	
Kashiwagi 2015E^28^	Type 2	Metformin	IPRA 50 mg	112	24	56.2	8.25	25.96	NR	4
			Placebo	56		57.7	8.38	25.47	NR	
Haneda 2016^29^	Type 2	Unclear	LUSEO 2.5 mg	95	24	67.9	7.72	25.45	52.0	4
			Placebo	50		68.4	7.69	25.81	52.4	
Seino 2014A^30^	Type 2	None	LUSEO 2.5 mg	79	24	58.9	8.14	25.98	NR	5
			Placebo	79		59.6	8.17	25.34	NR	
Seino 2014B^31^	Type 2	None	LUSEO 1 mg	55	12	58.5	7.77	24.51	NR	5
			LUSEO 2.5 mg	56		57.4	8.05	24.79	NR	
			LUSEO 5 mg	54		57.3	7.86	26.43	NR	
			LUSEO 10 mg	58		59.6	7.95	23.36	NR	
			Placebo	57		57.1	7.92	25.15	NR	
Seino 2014C^32^	Type 2	None	LUSEO 0.5 mg	60	12	55.2	8.16	25.4	NR	5
			LUSEO 2.5 mg	61		58.3	8.07	24.8	NR	
			LUSEO 5 mg	61		56.8	8.16	24.5	NR	
			Placebo	54		57.6	7.88	25.2	NR	
Kaku 2014^33^	Type 2	None	TOFO 10 mg	57	24	58.6	8.45	25.07	84.90	5
			TOFO 20 mg	58		56.6	8.34	24.99	86.78	
			TOFO 40 mg	58		57.0	8.37	25.78	86.00	
			Placebo	56		56.8	8.41	26.00	83.78	
Terauchi 2017^34^	Type 2	Insulin, DPP-4 inhibitor, rescue therapy	TOFO 20 mg	141	16	59.1	8.53	25.8	79.7	5
			Placebo	70		56.4	8.40	26.9	79.5	

*N* number of patients, *SGLT2* sodium-glucose co-transporter 2, *BMI* body mass index, *DM* diabetes mellitus, *DPP-4* dipeptidyl peptidase-4, *eGFR* estimated glomerular filtration rate, *CANA* canagliflozin, *DAPA* dapagliflozin, *EMPA* empagliflozin, *IPRA* ipragliflozin, *LUSEO* luseogliflozin, *TOFO* tofogliflozin, *NR* not reported.

*Data were extracted on week 24 before the up-titration of SGLT2 inhibitors was initiated.

^a^Data were means. ^b^Data were points.

### Quality assessment of each RCT

The Jadad scale^10^ of the studies examined ranged between 4 and 5 points (Table 1). We also assessed the risk of bias of RCTs based on the Cochrane handbook^11^. The majority of studies were high-quality RCTs. “Low risk” was the highest in the domains of the blinding of participants and personnel and the blinding of outcome assessments. “Unclear risk” was the highest in the domains of random sequence generation and allocation concealment. “High risk” was indicated in the definition of adverse events (other bias) (Supplementary Figure S2).

### Glycemic control

Twenty-two trials were included in the meta-analysis. Statistical heterogeneity was observed among trials (I^2^ = 90%). HbA1c values were significantly better with SGLT2 inhibitors than with a placebo [mean difference − 0.83 (95% CI − 0.96 to − 0.70) %, *p* < 0.00001], and all types of SGLT2 inhibitors showed a significant difference in the sub-group analysis. The IPRA group had the highest weight (31.5%), whereas the DAPA and TOFO groups had the lowest weight (9.3% each) (Supplementary Figure S3).

### Hypoglycemia

**Figure 1:**
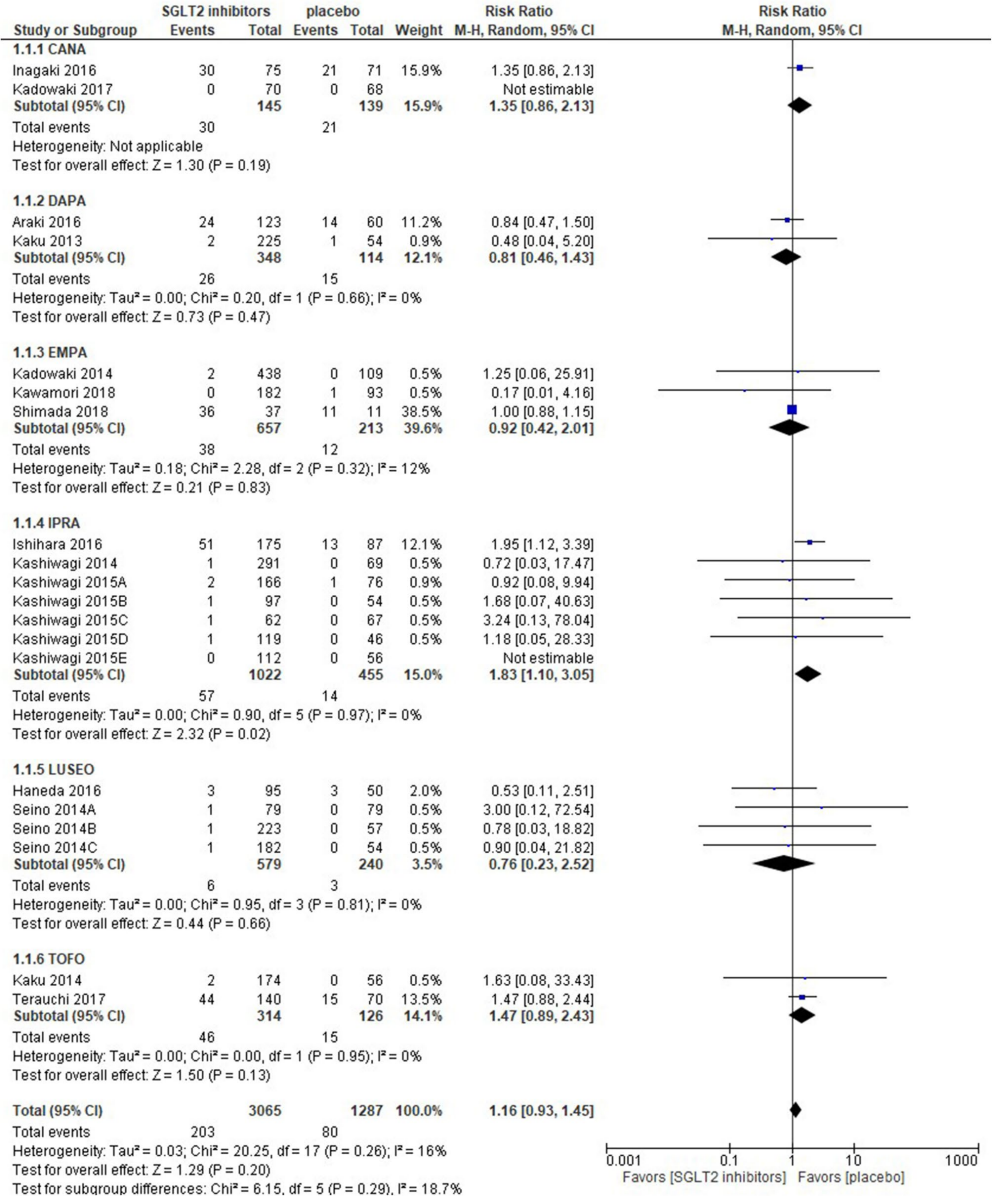
Forest plot for the risk of hypoglycemia. *CANA* canagliflozin, *DAPA* dapagliflozin, *EMPA* empagliflozin, *IPRA* ipragliflozin, *LUSEO* luseogliflozin, *TOFO* tofogliflozin, *SGLT2* sodium-glucose co-transporter 2.

Eighteen out of the 20 studies retrieved were quantified in the meta-analysis; 2 studies were unable to be quantified because of the lack of hypoglycemic events in both arms and were shown as “not estimable” in Fig. 1. SGLT2 inhibitors were associated with a similar risk of hypoglycemia as a placebo [risk ratio 1.16 (95% CI 0.93–1.45), *p* = 0.20], and the IPRA group showed a significant difference in the sub-group analysis. The EMPA group had the greatest weight (39.6%), whereas the LUSEO group had the lowest weight (3.5%). Statistical homogeneity was observed among trials (I^2^ = 16%) (Fig. 1).

### UTI

**Figure 2:**
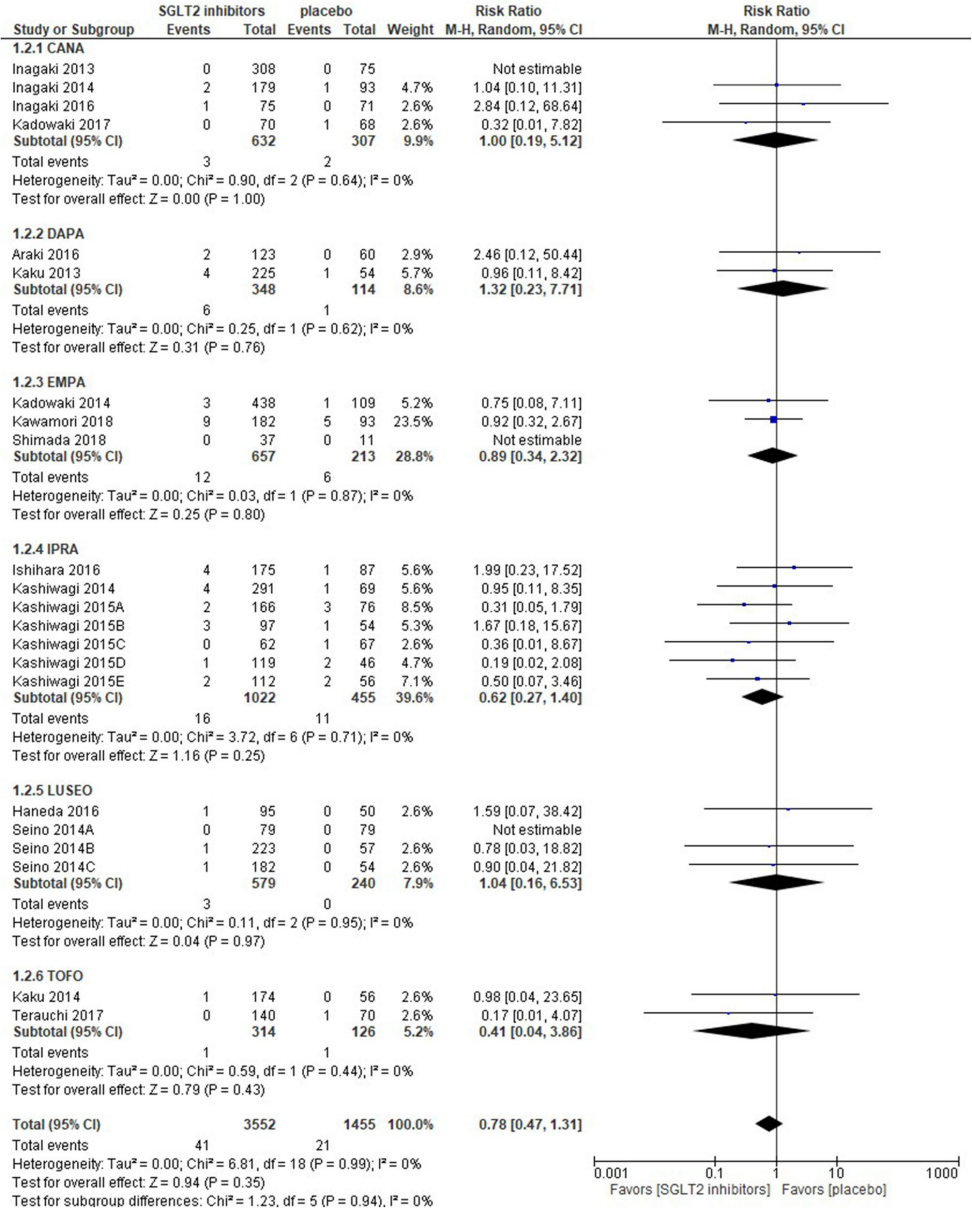
Forest plot for the risk of urinary tract infection. *CANA* canagliflozin, *DAPA* dapagliflozin, *EMPA* empagliflozin, *IPRA* ipragliflozin, *LUSEO* luseogliflozin, *TOFO* tofogliflozin, *SGLT2* sodium-glucose co-transporter 2.

Nineteen out of the 22 studies retrieved were quantified in the meta-analysis; 3 studies were unable to be quantified because of the lack of UTI events in both arms and were shown as “not estimable” in Fig. 2. SGLT2 inhibitors were associated with a similar risk of UTI as a placebo [risk ratio 0.78 (95% CI 0.47–1.31), *p* = 0.35], and no groups showed a significant difference in the sub-group analysis. The IPRA group had the greatest weight (39.6%), whereas the TOF group had the lowest weight (5.2%). Statistical homogeneity was observed among trials (I^2^ = 0%) (Fig. 2).

### Genital infection

**Figure 3:**
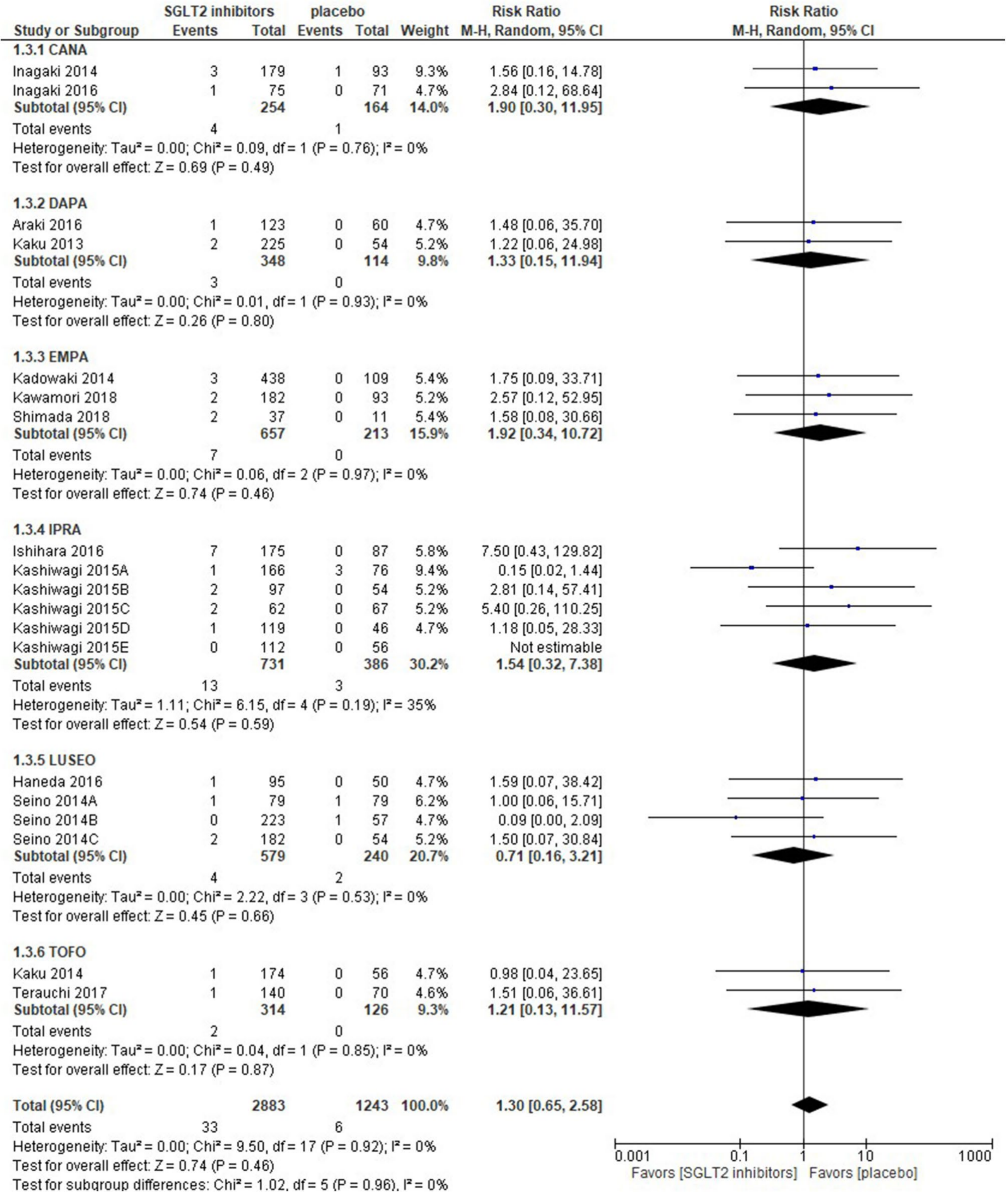
Forest plot for the risk of genital infection. *CANA* canagliflozin, *DAPA* dapagliflozin, *EMPA* empagliflozin, *IPRA* ipragliflozin, *LUSEO* luseogliflozin, *TOFO* tofogliflozin, *SGLT2* sodium-glucose co-transporter 2.

Eighteen out of the 19 studies retrieved were quantified in the meta-analysis; 1 study was unable to be quantified because of the lack of genital infection events in both arms and was shown as “not estimable” in Fig. 3. SGLT2 inhibitors were associated with a similar risk of genital infection as a placebo [risk ratio 1.30 (95% CI 0.65–2.58), *p* = 0.46], and no groups showed a significant difference in the sub-group analysis. The IPRA group had the greatest weight (30.2%), whereas the TOFO group had the lowest weight (9.3%). Statistical homogeneity was observed among trials (I^2^ = 0%) (Fig. 3).

### Hypovolemia

**Figure 4:**
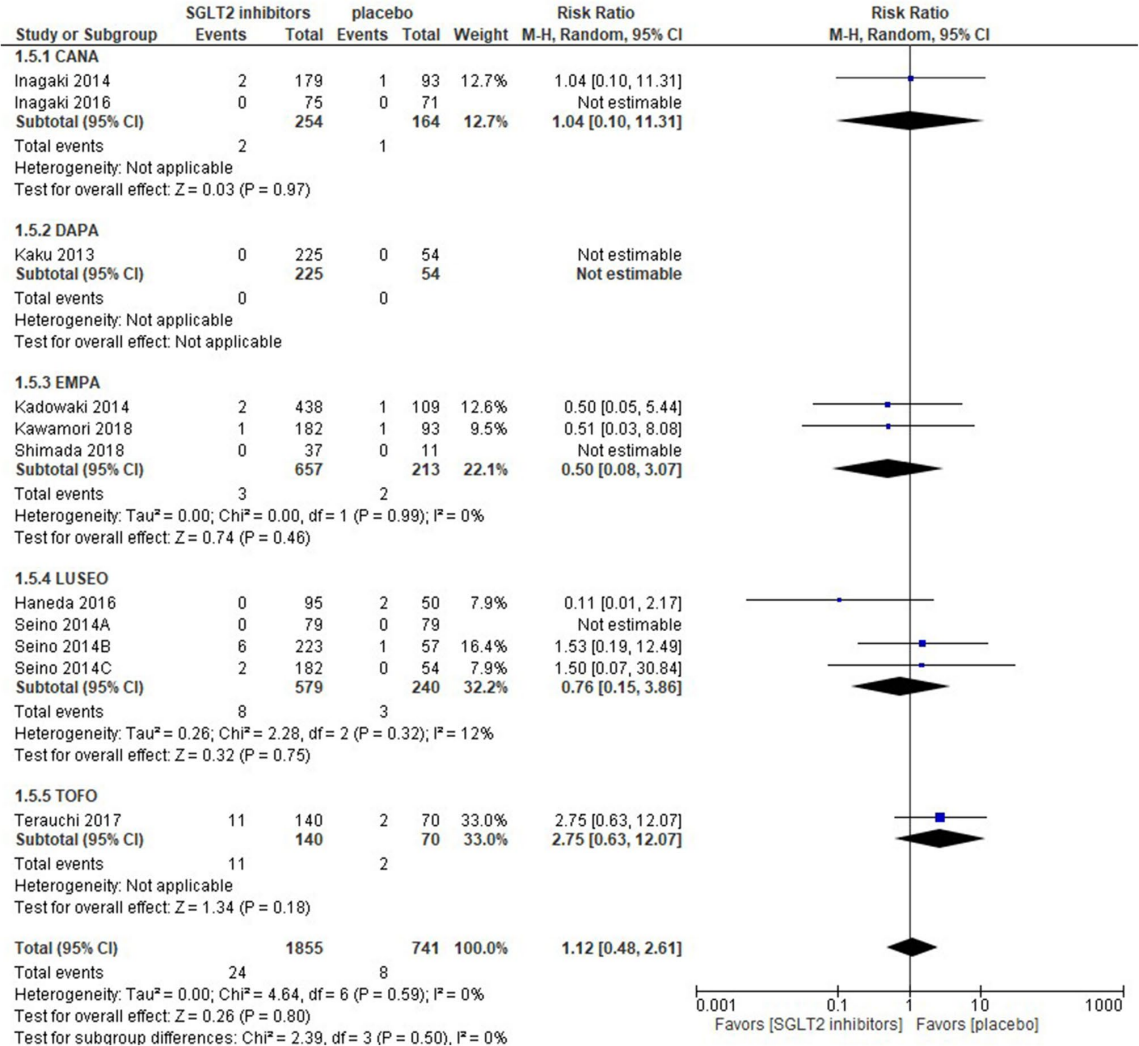
Forest plot for the risk of hypovolemia. *CANA* canagliflozin, *DAPA* dapagliflozin, *EMPA* empagliflozin, *LUSEO* luseogliflozin, *TOFO* tofogliflozin, *SGLT2* sodium-glucose co-transporter 2.

Seven out of the 11 studies retrieved were quantified in the meta-analysis; 4 studies were unable to be quantified because of the lack of hypovolemic events in both arms and were shown as “not estimable” in Fig. 4. SGLT2 inhibitors were associated with a similar risk of hypovolemia as a placebo [risk ratio 1.12 (95% CI 0.48–2.61), *p* = 0.80], and no groups showed a significant difference in the sub-group analysis. The LUSEO group had the greatest weight (32.2%), whereas the CANA group had the lowest weight (12.7%). Statistical homogeneity was observed among trials (I^2^ = 0%) (Fig. 4).

### Fracture

**Figure 5:**
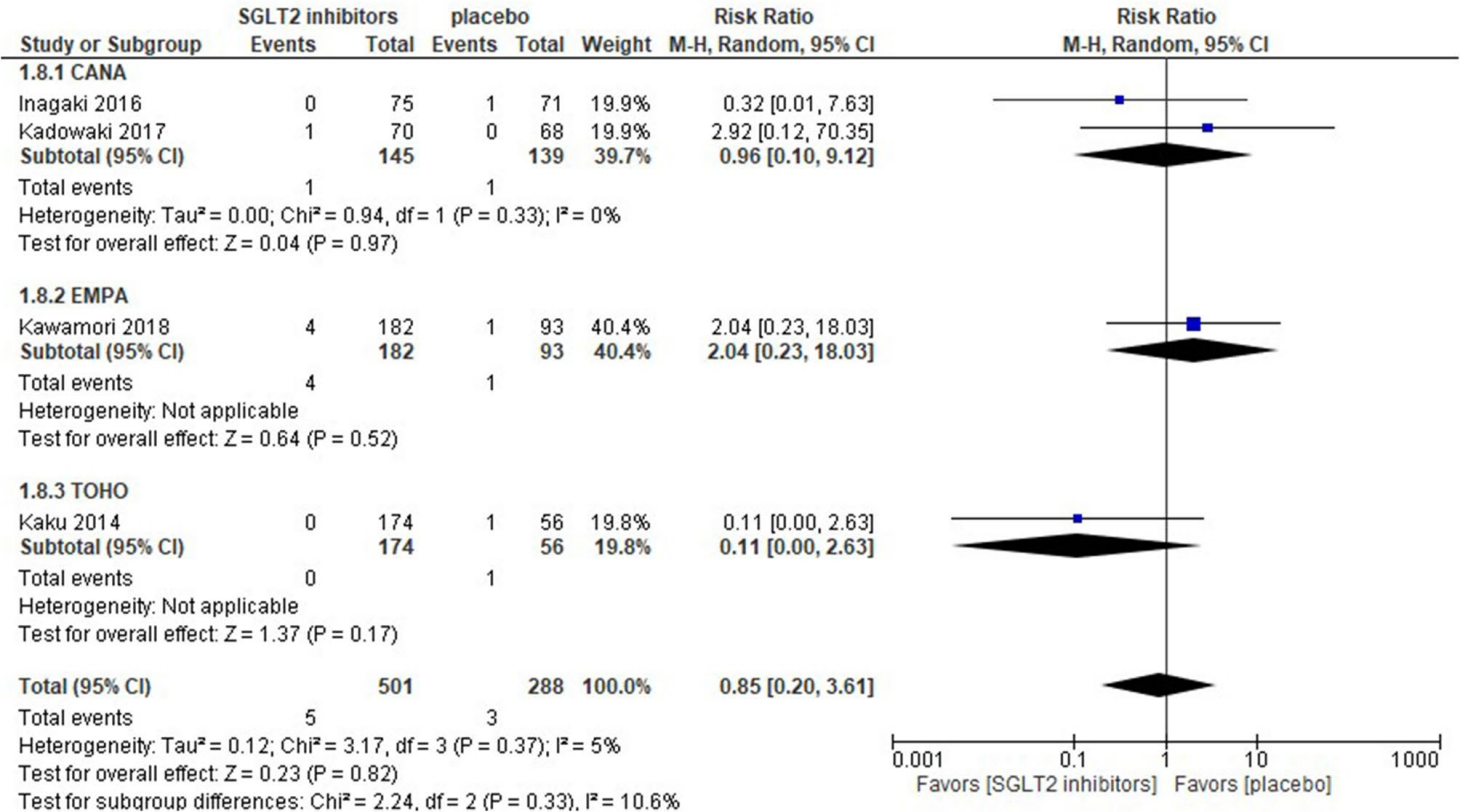
Forest plot for the risk of fracture. *CANA* canagliflozin, *EMPA* empagliflozin, *TOFO* tofogliflozin, *SGLT2* sodium-glucose co-transporter 2.

Four studies were quantified in the meta-analysis. Inagaki et al. reported 1 foot fracture and Kaku et al. reported 1 fibular fracture^15,33^. The remaining 2 studies did not report the fracture type. SGLT2 inhibitors were associated with a similar risk of fracture as a placebo [risk ratio 0.85 (95% CI 0.20–3.61), *p* = 0.82], and no groups showed a significant difference in the sub-group analysis. The EMPA group had the greatest weight (40.4%), whereas the TOFO group had the lowest weight (19.8%). Statistical homogeneity was observed among trials (I^2^ = 5%) (Fig. 5).

### Diabetic ketoacidosis

Two studies reported diabetic ketoacidosis^20,21^; however, we were unable to combine these data because neither study had any diabetic ketoacidosis events in either of their arms.

### Publication bias

Three outcomes (hypoglycemia, UTI, and genital infection) included more than 10 RCTs. Funnel plots visually detected a publication bias in all 3 outcomes (Supplementary Figures S4-6).

### Additional analyses

The sub-group analysis including only patients with type 2 DM showed that the risk of hypoglycemia was higher with SGLT2 inhibitors than with a placebo. This was not consistent with the results of the main analysis (Supplementary Table S1).

## Discussion

We herein conducted a systematic review and meta-analysis to summarize the available literature and appraise the safety profiles of SGLT2 inhibitors in Japanese patients with DM. The results obtained revealed that SGLT2 inhibitors were associated with similar risks of hypoglycemia, UTI, genital infection, hypovolemia, and fracture as a placebo. The safety data of the present analysis had negligible heterogeneity (I^2^ ≤ 18%).

The result showing that SGLT2 inhibitors had a similar risk of hypoglycemia as a placebo [risk ratio 1.16 (95% CI 0.93–1.45), I^2^ = 16%] was consistent with previous findings^36^; however, their data differed from the present study, which partially included patients with type 1 DM. Moreover, a SGLT2 inhibitor as monotherapy among Asian and non-Asian patients with type 2 DM did not increase the risk of hypoglycemia^7,36^. The addition of combination therapies to an oral hypoglycemic agent(s) or insulin is known to generally increase the risk of hypoglycemia; however, multiple meta-analyses including patients with type 1 DM and with no restrictions in races/ethnicities revealed that even dual combination therapy with a SGLT2 inhibitor and insulin did not increase the risk of hypoglycemia over that with a placebo^2,3,37^. This may be attributed to the insulin-independent anti-hyperglycemic effects of SGLT2 inhibitors rather than racial or ethnic differences.

The present study demonstrated that SGLT2 inhibitors had a similar risk of UTI [risk ratio 0.78 (95% CI 0.47–1.31)] as a placebo. This result supports the findings of 2 previous studies including Asian patients with type 2 DM^7,36^. Furthermore, a larger meta-analysis of more than 100 RCTs and with no racial or ethnic restrictions showed that the risk of UTI was similar between SGLT2 inhibitors and a placebo^38^. A large population-based cohort study using U.S. databases of patients with employer-based insurance also reported that in comparisons with glucagon-like peptide-1 receptor agonists, treatments with SGLT2 inhibitors were not associated with the risks of both severe and non-severe UTI^39^. These findings suggest that SGLT2 inhibitors are unlikely to increase the risk of UTI regardless of whether patients are Asians or non-Asians. Two previous meta-analyses of Asian populations showed that SGLT2 inhibitors consistently increased the risk of genital infection^7,36^. Furthermore, a few meta-analyses with long-term follow-ups reported an increased risk of genital infection with SGLT2 inhibitors^40,41^. One possible explanation for the inconsistency between our results and these findings is that the RCTs retrieved had relatively short-term follow-ups (at most 24 weeks). Three meta-analyses consistently showed that a treatment with DAPA may dose-dependently increase the risk of UTI and genital infection^38,40,42^; however, DAPA did not increase the risk of either event in sub-analyses (Figs. 2, 3).

The present review showed that in comparison with a placebo, SGLT2 inhibitors had a similar risk of hypovolemia [risk ratio 1.12 (95% CI 0.48–2.61)]. A previous study on East Asian patients with type 2 DM found no significant difference in the risk of hypotension between SGLT2 inhibitors and a placebo^36^. In contrast, one RCT with a long-term follow-up of more than 100 weeks among mainly Caucasian patients with type 2 DM showed that the prevalence of volume depletion-related adverse events was threefold higher with SGLT2 inhibitors than with a placebo^4^. Since they reported that these events with SGLT2 inhibitors generally occurred within 26 weeks and that a longer exposure to SGLT2 inhibitors may have resulted in a higher incidence of these events^4^, the incidence of hypovolemia in a short-term follow-up may be lower among Japanese patients treated with SGLT2 inhibitors than among Caucasian patients. However, the present results need to be interpreted with caution because the definition of hypovolemia or volume depletion varied among the studies retrieved. Moreover, a meta-analysis of patients with type 2 DM and chronic kidney disease showed a slightly elevated risk of hypovolemia with SGLT2 inhibitors^43^. Further studies with a standardized definition of adverse events and involving more diverse populations are needed to support the present results.

The present analysis indicated that SGLT2 inhibitors were associated with a similar risk of fracture as a placebo [risk ratio 0.85 (95% CI 0.20–3.61)] (Fig. 5). This was consistent with a meta-analysis of East Asian patients^36^ and with a network meta-analysis including approximately 80% Caucasian patients^44^; however, a sub-analysis of the network meta-analysis showed the opposite findings, namely, Asian populations had a slightly higher risk of fracture^44^. The reason for this disparity is unclear. The longer duration of treatment with SGLT2 inhibitors was associated with higher risk of fracture^45^. Cohort or case–control studies rather than RCTs with short-term durations are generally more likely to show long-term or rare adverse events. Therefore, the duration of the follow-up in our analysis was too short to assess the risk of fracture; previous reports that evaluated the risk of fracture had the same limitation as our analysis^36,44,46^. Additionally, our fracture outcome did not include all types of SGLT2 inhibitors. Collectively, our results regarding fracture risk along with previous findings indicate that more RCTs with long-term follow-ups and individual SGLT2 inhibitors are needed in the future.

A meta-analysis has not yet been conducted on the risk of diabetic ketoacidosis in Japanese DM patients treated with SGLT2 inhibitors. Two previous meta-analyses of Asian populations also did not examine this event^7,36^. We found two RCTs that reported diabetic ketoacidosis in Japanese populations^20,21^; however, we were unable to quantify these data because neither study had cases in both arms. The findings obtained showed that EMPA was unlikely to increase the risk of diabetic ketoacidosis in Japanese DM patients. Previous studies demonstrated that EMPA dose-dependently, but modestly, increased the levels of ketone bodies in Japanese patients with type 1 and 2 DM^21,47^; however, since there has only been 1 RCT each on Japanese patients with type 1 and 2 DM^20,21^, further RCTs that include Japanese patients with different types of DM are needed to quantify the risk of diabetic ketoacidosis.

Our sub-analysis including only type 2 DM indicated that the risk of hypoglycemia was higher with SGLT2 inhibitors than with a placebo [RR 1.30 (95% CI 1.01–1.65)] (Supplementary Table S1). This result was partially in line with the findings of an earlier meta-analysis of Asian patients with type 2 DM^7^. Since the excluded RCT^21^ had the shortest study duration of 4 weeks and the greatest weight of 38.5% in the hypoglycemia outcome (Fig. 1), the RCT may have affected this result.

The present study has some strengths. To the best of our knowledge, this is the first systematic literature review and meta-analysis to appraise the safety profiles of SGLT2 inhibitors in Japanese patients with DM. Furthermore, the safety data of our analyses consistently had negligible heterogeneity (I^2^ ≤ 18%) and the majority of the studies retrieved were high-quality RCTs (Supplementary Figure S2). However, the present study also had some limitations. It may have had a publication bias because we only retrieved published studies. We were unable to rule out the impact of anti-hyperglycemic agents or to exclude type 1 DM patients; the former is because some studies included patients who were treated with an oral hypoglycemic agent or insulin, while the latter is due to 1 RCT including patients with type 1 DM^21^. Therefore, we were only able to evaluate the safety profiles of SGLT2 inhibitors in all Japanese patients with DM; however, we confirmed that the results of the sub-analysis of patients with type 2 DM only were consistent with those of the main analyses among all patients with DM (Supplementary Table S1). Other limitations are that the RCTs retrieved did not always set the adverse events that we evaluated as their primary endpoint, and also that the numbers of different types of SGLT2 inhibitors pooled were unbalanced. Therefore, our data may be biased.

## Conclusion

The present results suggest that in comparison with a placebo, SGLT2 inhibitors were associated with similar risks of hypoglycemia, UTI, genital infection, hypovolemia, and fracture. Treatment with SGLT2 inhibitors among Japanese patients with DM suggests favorable safety profiles. However, further evidence from studies with a longer duration, involving more diverse populations, such as patients with different types of DM, or including individual SGLT2 inhibitors is needed to resolve the limitations of the present study. We consider the present results to be informative for SGLT2 inhibitors users with concerns regarding the safety profiles of SGLT2 inhibitors.

## Supplementary Information

Supplementary Information 1.

## Data Availability

The datasets used and/or analyzed during the present study are available from the corresponding author upon reasonable request.
