# Supplementary material for: A literature review and meta-analysis of safety profiles of SGLT2 inhibitors in Japanese patients with diabetes mellitus
Source: Sci Rep. 2021 Jun 29;11:13472. doi: 10.1038/s41598-021-92925-2 (PMC8241876; doi:10.1038/s41598-021-92925-2)
Supplement: Supplementary file 1 — Supplementary Information 1. [file 41598_2021_92925_MOESM1_ESM.docx]

Supplementary information

A literature review and meta-analysis of safety profiles of SGLT2 inhibitors in Japanese patients with diabetes mellitus

Junichi Mukai, Shinya Kanno, and Rie Kubota

| Supplementary Table S1 Summary of subgroup analyses | | | | | | | | |
| --- | --- | --- | --- | --- | --- | --- | --- | --- |
|  | Outcome | Trial,  n | Reference(s) excluded | SGLT2 inhibitors, n | Placebo,  n | Risk ratio  [95%CI] | I^2^  (%) | *p* |
| Only patients with type 2 DM | Hypoglycemia | 19 | Shimada 2018 ^21^ | 3028 | 1276 | 1.30 [1.01, 1.65] | 0 | 0.04 |
|  | UTI | 21 | Shimada 2018 ^21^ | 3515 | 1444 | 0.78 [0.47, 1.31] | 0 | 0.35 |
|  | Genital infection | 17 | Shimada 2018 ^21^ | 2846 | 1232 | 1.28 [0.63, 2.60] | 0 | 0.49 |
|  | Hypovolemia | 10 | Shimada2018 ^21^ | 1518 | 605 | 1.12 [0.48, 2.61] | 0 | 0.80 |
|  | Fracture | NA |  |  |  |  |  |  |
| Only patients treated with a SGLT2 inhibitor  as monotherapy | Hypoglycemia | 9 | Inagaki 2016 ^15^, Kadowaki 2017 ^16^, Araki 2016 ^17^, Kawamori 2018 ^20^, Shimada 2018 ^21^, Ishihara 2016 ^22^, Kashiwagi 2015A ^24^, Kashiwagi 2015B ^25^, Kashiwagi 2015D ^27^, Kashiwagi 2015E ^28^, Terauchi 2017 ^34^ | 1769 | 595 | 0.89 [0.37, 2.13] | 0 | 0.79 |
|  | UTI | 11 | Inagaki 2016 ^15^, Kadowaki 2017^16^, Araki 2016 ^17^, Kawamori 2018 ^20^, Shimada 2018 ^21^, Ishihara 2016 ^22^, Kashiwagi 2015A ^24^, Kashiwagi 2015B ^25^, Kashiwagi 2015D ^27^,  Kashiwagi 2015E ^28^, Terauchi 2017 ^34^ | 2256 | 763 | 0.88 [0.37, 2.13] | 0 | 0.78 |
|  | Genital infection | 9 | Inagaki 2016 ^15^,  Araki 2016 ^17^, Kawamori 2018 ^20^, Shimada 2018 ^21^, Ishihara 2016 ^22^, Kashiwagi 2015A ^24^, Kashiwagi 2015B ^25^, Kashiwagi 2015D ^27^, Kashiwagi 2015E ^28^, Terauchi 2017 ^34^ | 1657 | 619 | 1.21 [0.46, 3.19] | 0 | 0.7 |
|  | Hypovolemia | 8 | Kawamori 2018 ^20^, Shimada 2018 ^21^, Terauchi 2017 ^34^ | 1196 | 442 | 0.76 [0.25, 2.33] | 0 | 0.63 |
|  | Fracture | 3 | Kawamori 2018 ^20^ | 319 | 195 | 0.46 [0.07, 3.12] | 7 | 0.43 |
| DM diabetes mellitus SGLT2 sodium-glucose co-transporter 2, UTI urinary tract infection, NA not applicable, I^2^ heterogeneity | | | | | | | | |


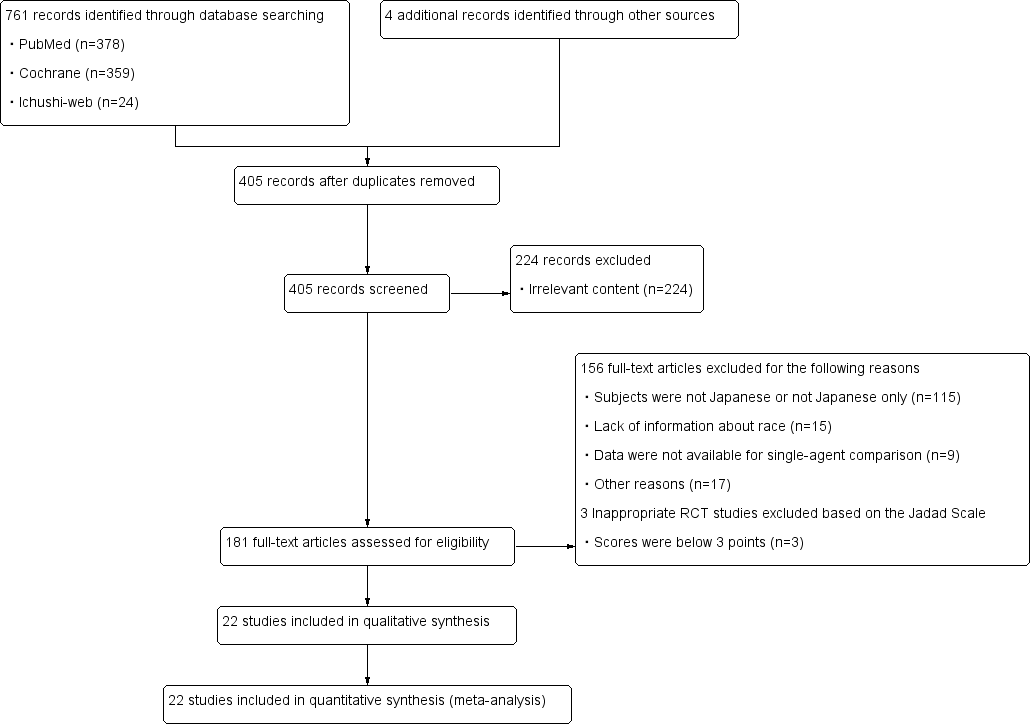


Supplementary Figure S1 Process to identify eligible randomized controlled trials following PRISMA


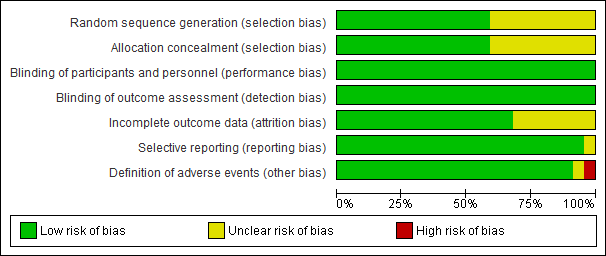


Supplementary Figure S2 Risk of bias graph of 22 randomized controlled trials


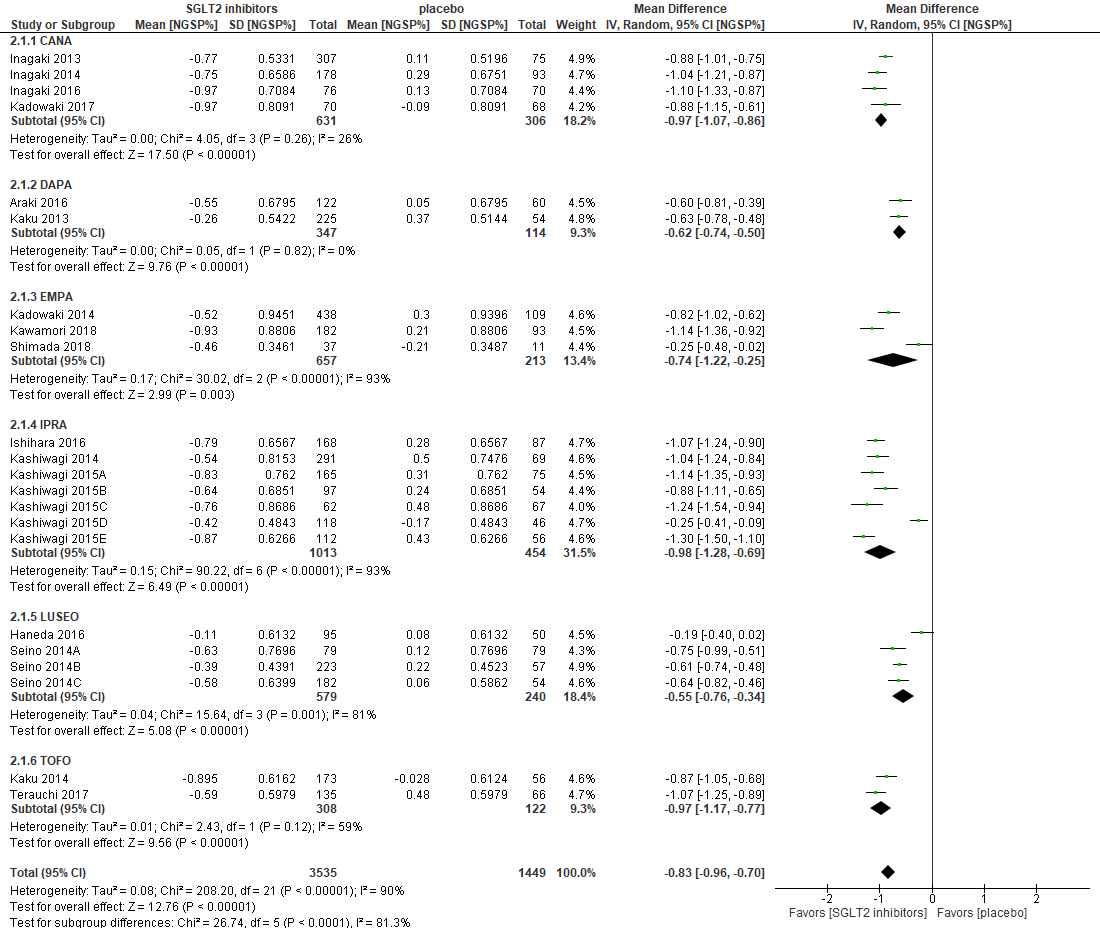


Supplementary Figure S3 Relationship between SGLT2 inhibitors and changes in HbA1c

Abbreviations: CANA, canagliflozin; DAPA, dapagliflozin; EMPA, empagliflozin; IPRA, ipragliflozin; LUSEO, luseogliflozin; TOFO, tofogliflozin; SGLT2, sodium-glucose co-transporter 2.


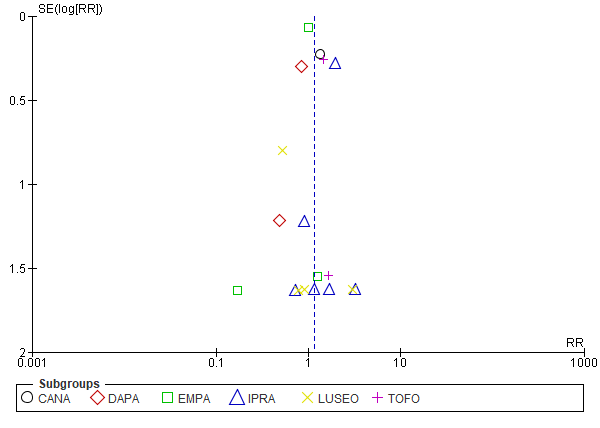


Supplementary Figure S4 Funnel plot for the risk of hypoglycemia

Abbreviations: CANA, canagliflozin; DAPA, dapagliflozin; EMPA, empagliflozin; IPRA, ipragliflozin; LUSEO, luseogliflozin; TOFO, tofogliflozin; SE, standard error; RR; risk ratio.


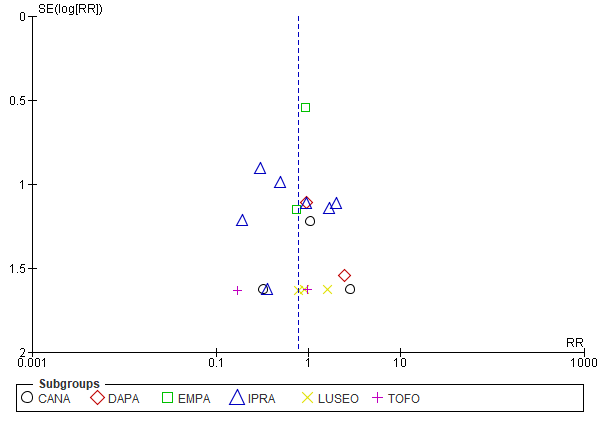


Supplementary Figure S5 Funnel plot for the risk of urinary tract infection

Abbreviations: CANA, canagliflozin; DAPA, dapagliflozin; EMPA, empagliflozin; IPRA, ipragliflozin; LUSEO, luseogliflozin; TOFO, tofogliflozin; SE, standard error; RR; risk ratio.


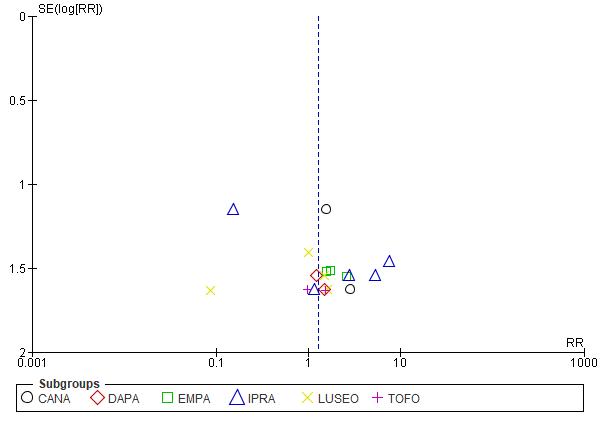


Supplementary Figure S6 Funnel plot for the risk of genital infection

Abbreviations: CANA, canagliflozin; DAPA, dapagliflozin; EMPA, empagliflozin; IPRA, ipragliflozin; LUSEO, luseogliflozin; TOFO, tofogliflozin; SE, standard error; RR; risk ratio.
